# A New Ibuprofen Derivative Inhibits Platelet Aggregation and ROS Mediated Platelet Apoptosis

**DOI:** 10.1371/journal.pone.0107182

**Published:** 2014-09-19

**Authors:** Kodagahalli S. Rakesh, Swamy Jagadish, Ajjampura C. Vinayaka, Mahadevappa Hemshekhar, Manoj Paul, Ram M. Thushara, Mahalingam S. Sundaram, Toreshettahally R. Swaroop, Chakrabhavi D. Mohan, Basappa, Marilinganadoddi P. Sadashiva, Kempaiah Kemparaju, Kesturu S. Girish, Kanchugarakoppal S. Rangappa

**Affiliations:** 1 DOS in Chemistry, University of Mysore, Manasagangotri, Mysore, India; 2 DOS in Biochemistry, University of Mysore, Manasagangotri, Mysore, India; 3 Laboratory of Chemical Biology, Department of Chemistry, Bangalore University, Bangalore, India; 4 Department of Studies and Research in Biochemistry, Tumkur University, Tumkur, India; Royal College of Surgeons, Ireland

## Abstract

Thrombocytopenia is a serious issue connected with the pathogenesis of several human diseases including chronic inflammation, arthritis, Alzheimer's disease, cardiovascular diseases (CVDs) and other oxidative stress-associated pathologies. The indiscriminate use of antibiotics and other biological drugs are reported to result in thrombocytopenia, which is often neglected during the treatment regime. In addition, augmented oxidative stress induced by drugs and pathological conditions has also been shown to induce thrombocytopenia, which seems to be the most obvious consequence of elevated rate of platelet apoptosis. Thus, blocking oxidative stress-induced platelet apoptosis would be of prime importance in order to negotiate thrombocytopenia and associated human pathologies. The current study presents the synthesis and platelet protective nature of novel ibuprofen derivatives. The potent anti-oxidant ibuprofen derivative 4f was selected for the study and the platelet protective efficacy and platelet aggregation inhibitory property has been demonstrated. The compound 4f dose dependently mitigates the oxidative stress-induced platelet apoptosis in both platelet rich plasma and washed platelets. The platelet protective nature of compound 4f was determined by assessing various apoptotic markers such as ROS generation, cytosolic Ca^2+^ levels, PS externalization, cytochrome C translocation, Caspase activation, mitochondrial membrane depolarization, cytotoxicity, LDH leakage and tyrosine phosphorylation of cytosolic proteins. Furthermore, compound 4f dose dependently ameliorated agonist induced platelet aggregation. Therefore, compound 4f can be estimated as a potential candidate in the treatment regime of pathological disorders associated with platelet activation and apoptosis. In addition, compound 4f can be used as an auxiliary therapeutic agent in pathologies associated with thrombocytopenia.

## Introduction

Several human diseases including chronic inflammation, diabetes, arthritis, cardiovascular diseases (CVDs) and other oxidative stress-associated pathologies are also being linked to mean platelet volume and reduced platelet count, in other words thrombocytopenia [1]–[4]. The augmented oxidative stress as evidenced by elevated reactive oxygen species (ROS) is shown to induce thrombocytopenia, which seems to be the most obvious consequence of elevated rate of platelet apoptosis [5]. Platelets are simple anuclear cells, yet execute a plethora of physiological actions such as hemostasis, thrombosis and wound healing [6]. It is no more surprising that the anuclear platelets end their life through apoptosis like any other nucleated cell. Several studies have demonstrated that platelets possess the necessary cellular machinery to undergo apoptosis [7], [8]. Besides aging, several other factors like chemical and physical agonists, oxidative stress-induced pathological conditions are also shown to trigger apoptosis in platelets [5], [9], [10]. They undergo apoptosis *via* intrinsic apoptotic pathway; however, very few studies have reported the extrinsic pathway too [5], [11]. The altered platelet number and functions would certainly lead to bleeding disorders and thrombotic diseases. The distorted platelet functions are also responsible for the multifactorial diseases including coronary heart disease (CHD) and other cardiovascular diseases (CVDs), inflammatory and immune reactions [12]–[14].

The cytoplasmic events of intrinsic apoptotic pathway is instigated by elevated endogenous reactive oxygen species (ROS) particularly, hydrogen peroxide (H_2_O_2_). The principal target of oxidative stress is mitochondrial damage resulting in the down-regulation of electron transport chain, which in turn increases ROS generation and formation of mitochondrial permeability transition pore (MPTP). This would significantly induce depolarization of mitochondrial membrane potential (ΔΨ*m*) causing leakage of cytochrome c (cyt c) into the cytoplasm. Furthermore, the leaked cyt c in association with pro-apoptotic factors like Apoptotic Protease Activating Factor-1 (Apaf-1) mediate the activation of caspase-9 (elicitor caspase), which in turn activates caspase-3 (executioner caspase). Finally, phosphatidylserine (PS) externalization occurs, which is a signal for phagocytosis [7], [8], [15]. Besides, the process of platelet apoptosis also releases PS-positive membrane fractions called microparticles (MPs), which significantly contribute to the pathogenesis of atherosclerosis, central nervous system damage and neoplasia [16].

Thus, blocking oxidative stress-induced platelet apoptosis would be of prime importance in order to combat thrombocytopenia and associated human pathologies. Hence, there is a need for potent molecules that can protect platelets from the premature death. Till date there are only two reports describing the inhibition of platelet apoptosis by phytochemicals namely, cinnamtannin-B1 and crocin [17], [18]. Both the molecules ameliorate the oxidative stress-induced platelet apoptosis by modulating ROS mediated mitochondrial damage. Ibuprofen is a non-steroidal anti-inflammatory drug (NSAID) used to treat the pathological conditions related to inflammation. It exerts its anti-inflammatory action by blocking cyclooxygenase-1 non-specifically and thereby thromboxane production [19]. In the recent past, studies reported that Ibuprofen augments the anticancer activity of cisplatin in lung adenocarcinoma cells by blocking heat shock protein 70 [20]. However, laboratory testing of drug-induced immune thrombocytopenia (DIIT) reported development of immune thrombocytopenia by ibuprofen [21]. Based on these facts, a series of novel ibuprofen derivatives 4(a-f) are synthesized by modifying the functional groups and evaluated for improved anti-oxidant activity. Among the derivatives, the potent anti-oxidant compound 4f was investigated for the protective efficacy against platelet apoptosis as a clinical implication to thrombocytopenia.

## Materials and Methods

### Chemicals/Reagents

Calcium ionophore (A23187), benzamidine hydrochloride, N-acetyl-Leu-Glu-His-Asp trifluoro methylcoumarin (AC-LEHD-FMC), acetyl-Asp-Glu-Val-Asp-7-amido-4-methylcoumarin (AC-DEVD-AMC), sodium *ortho*vanadate, dimethyl sulfoxide (DMSO), fluorescein isothiocyanate (FITC)-labeled annexin V, 5-(and-6)-chloromethyl-2′,7′-dichlorodihydrofluorescein diacetate acetylester (CM-H2DCFDA), CHAPS, rhodamine 123, leupeptin hydrochoride, N-(2-Hydroxyethyl) piperazine-*N*′-ethanesulfonic acid (HEPES), fura-2/AM, monoclonal anti-phosphotyrosine antibody, acridine orange 10-nonyl bromide (NAO) and dithiothreitol (DTT) were from Sigma Chemicals, St. Louis (USA). Monoclonal anti-cytochrome c antibody and anti-β-actin were from Epitomics Burlingame, CA (USA). Anti-Caspase-3 antibody was from Santa Cruz Biotechnology, Inc. Texas (USA). Collagen type-I was from Chrono-log Corporation, Pennsylvania (USA). 3-(4,5-dimethylthiazol-2-yl)-2,5-diphenyltetrazolium bromide (MTT) and 1, 1-diphenyl-2-picrylhdrazyl (DPPH) were from HiMedia Laboratories, Mumbai (India). Lactate dehydrogenase (LDH) kit was from AGAPPE diagnostics Ltd., Kerala (India). γ-glutamyl *p*-nitroanilide and glycylglycine were from Sisco Research laboratories Pvt Ltd., Mumbai (India). All other reagents were of analytical grade.

### Synthesis and characterization of Ibuprofen derivatives

The starting materials were commercially available and used as received without further purification. Reactions were monitored by TLC using precoated sheets of silica gel (G/UV-254) of 0.25 mm thickness (Merck 60F254) using UV light for visualization. The melting points were determined on Selaco melting point apparatus and are uncorrected. ^1^H and ^13^C NMR spectra were recorded on an NMR spectrometer operating at 400 and 100 MHz, respectively, using the residual solvent peaks as reference relative to SiMe_4_. Mass spectra were recorded using electrospray ionization (ESI) mass spectrometry. The C, H, and N analysis were performed using CE-400 CHN analyzer. Infrared spectra were recorded on Shimadzu FT-IR model 8300 spectrophotometer.

### Ethyl 2-[4-(2-methylpropyl)phenyl]propanoate (2)

A solution of Ibuprofen 1 (2 g, 9.6 mmol) and 0.5 mL concentrated sulfuric acid in ethanol (20 mL) was refluxed for 6 h. The solvent was removed under reduced pressure and the residue was taken up in CHCl_3_ (30 mL). The solution was extracted with saturated aqueous NaHCO_3_ solution (2×15 mL) and water. The organic layer was dried over anhydrous Na_2_SO_4_, filtered and solvent was evaporated under reduced pressure, affording 2 (2.1 g, 95%) as a clear oil.

### 2-[4-(2-methylpropyl)phenyl]propanehydrazide (3)

The mixture of ethyl ester of Ibuprofen 2 (2 g, 8.5 mmol) and hydrazine hydrate (0.8 mL, 16 mmol) in absolute ethanol (30 mL) was refluxed for 10 h. The solvent was removed under reduced pressure and the residue was added to ice cold water. The solid separated was filtered, washed and dried. The solid was purified by recrystallization from ethanol to get pure product 3 (1.7 g, 96%) as a white solid.

### 2-[4-(2-methylpropyl)phenyl]-*N*'-(phenylsulfonyl)propanehydrazide (4a-f)

To a stirred solution of 2-[4-(2-methylpropyl)phenyl]propanehydrazide (0.15 g, 0.68 mmol), substituted benzene sulfonyl chloride (0.68 mmol) in dichlorormethane (5 mL), triethylamine (0.14 mL, 1.0 mmol) was added at 0°C. The reaction mixture was brought to room temperature and further stirred for 2 h. After the completion of reaction, the reaction mixture was extracted with ethyl acetate (2×10 mL). The combined organic extracts were washed with water (3×5 mL), brine (1×10 mL) and dried over anhydrous Na_2_SO_4_, filtered and solvent was evaporated under reduced pressure to get crude products 4 (a-f), which were purified by column chromatography over silica gel using hexanes-EtOAc as eluent.

### 2-[4-(2-methylpropyl)phenyl]-*N*'-(phenylsulfonyl)propanehydrazide (4a)

Yield 89%, white solid; MP 112–114°C; IR (KBr) ν∼3320, 2951, 1675, 1585, 1124; ^1^H NMR (400 MHz, CDCl_3_) δ 7.82 (d, J = 6.4 Hz, 1H, NH), 7.72–7.57 (m, 5H, Ar-H), 7.21 (d, J = 6.4 Hz, 1H, NH), 7.07 (d, J = 7.6 Hz, 2H, Ar-H), 7.01 (d, J = 8.4 Hz, 2H, Ar-H), 3.42 (q, J = 6.1 Hz, 1H, CH), 2.44 (d, J = 7.6 Hz, 2H, ArCH_2_), 1.85 (m, 1H, CH), 1.26 (d, J = 7.2 Hz, 3H, Me), 0.88 (dd, J = 6.6 Hz, 1.0 Hz, 6H, Me_2_); ^13^C NMR (100 MHz, CDCl_3_) δ 177.8, 141.2, 136.4, 132.4, 130.8, 129.6, 127.2, 126.9, 125.6, 44.9, 44.4, 30.2, 22.4, 22.3, 17.9; Anal. Calcd for C_19_H_24_N_2_O_3_S: C 63.31, H 6.71, N 7.77. Found: C 63.32, H 6.72, N 7.78.

### *N*'-[(4-fluorophenyl)sulfonyl]-2-[4-(2-methylpropyl) phenyl]propanehydrazide (4b)

Yield 91%, white solid, MP 121–123°C; IR (KBr) ν∼3312, 2960, 1679, 1592, 1139; ^1^H NMR (400 MHz, CDCl_3_) δ 7.85 (d, J = 6.2 Hz, 1H, NH), 7.79 (d, J = 7.8 Hz, 2H, Ar-H), 7.62 (d, J = 7.8 Hz, 2H, Ar-H), 7.26 (d, J = 6.2 Hz, 1H, NH), 7.07 (d, J = 7.6 Hz, 2H, Ar-H), 7.01 (d, J = 8.4 Hz, 2H, Ar-H), 3.42 (q, J = 6.1 Hz, 1H, CH), 2.44 (d, J = 7.6 Hz, 2H, ArCH_2_), 1.85 (m, 1H, CH), 1.26 (d, J = 7.2 Hz, 3H, Me), 0.88 (dd, J = 6.6 Hz, 1.0 Hz, 6H, Me_2_); ^13^C NMR (100 MHz, CDCl_3_) δ 177.8, 168.8, 141.2, 136.4, 131.8, 129.6, 128.4, 127.2, 117.3, 44.9, 44.4, 30.2, 22.4, 22.3, 17.9; Anal. Calcd for C_19_H_23_FN_2_O_3_S: C 60.30, H 6.13, N 7.40. Found: C 60.31, H 6.15, N 7.43.

### *N*'-[(4-bromophenyl)sulfonyl]-2-[4-(2-methylpropyl)phenyl]propanehydrazide (4c)

Yield 81%, red solid, MP 116–118°C; IR (KBr) ν∼3340, 2949, 1668, 1598, 1162; ^1^H NMR (400 MHz, CDCl_3_) δ 7.85 (d, J = 6.2 Hz, 1H, NH), 7.72 (d, 2H, J = 7.2 Hz, Ar-H), 7.68 (d, 2H, J = 7.2 Hz, Ar-H), 7.25 (d, J = 6.2 Hz, 1H, NH), 7.07 (d, J = 7.6 Hz, 2H, Ar-H), 7.01 (d, J = 8.4 Hz, 2H, Ar-H), 3.42 (q, J = 6.1 Hz, 1H, CH), 2.44 (d, J = 7.6 Hz, 2H, ArCH_2_), 1.85 (m, 1H, CH), 1.26 (d, J = 7.2 Hz, 3H, Me), 0.88 (dd, J = 6.6 Hz, 1.0 Hz, 6H, Me_2_); ^13^C NMR (100 MHz, CDCl_3_) δ 177.6, 141.1, 135.8, 131.1, 130.4, 129.6, 128.3, 127.1, 126.9, 44.9, 44.4, 30.2, 22.4, 22.3, 17.9; Anal. Calcd for C_19_H_23_BrN_2_O_3_S: C 51.94, H 5.28, N 6.38. Found: C 51.95, H 5.31, N 6.39.

### *N*'-[(4-nitrophenyl)sulfonyl]-2-[4-(2-methylpropyl)phenyl]propanehydrazide (4d)

Yield 78%, yellow solid, MP 142-144°C; IR (KBr) ν∼3319, 2971, 1678, 1582, 1145; ^1^H NMR (400 MHz, CDCl_3_) δ 7.88 (d, J = 6.2 Hz, 1H, NH), 7.71–7.63 (m, 4H, Ar-H), 7.29 (d, J = 6.2 Hz, 1H, NH), 7.07 (d, J = 7.6 Hz, 2H, Ar-H), 7.01 (d, J = 8.4 Hz, 2H, Ar-H), 3.40 (q, J = 6.4 Hz, 1H, CH), 2.44 (d, J = 7.6 Hz, 2H, ArCH_2_), 1.85 (m, 1H, CH), 1.28 (d, J = 7.2 Hz, 3H, Me), 0.88 (dd, J = 6.6 Hz, 1.0 Hz,6H, Me_2_); ^13^C NMR (100 MHz, CDCl_3_) δ 177.8, 152.8, 144.1, 141.2, 136.4, 129.6, 128.6, 127.2, 125.1, 44.9, 44.4, 30.2, 22.4, 22.3, 17.9; Anal. Calcd for C_19_H_23_N_3_O_5_S: C 56.28, H 5.72, N 10.36. Found: C 56.29, H 5.74, N 10.38.

### *N*'-[(2,5-dichlorophenyl)sulfonyl]-2-[4-(2-methylpropyl)phenyl]propanehydrazide (4e)

Yield 85%, white solid, MP 102–104°C; IR (KBr) ν∼3364, 2983, 1658, 1518, 1084; ^1^H NMR (400 MHz, CDCl_3_) δ 7.82 (d, J = 6.4 Hz, 1H, NH), 7.90 (s, 1H, Ar-H), 7.71 (d, J = 8.0 Hz, 1H, Ar-H), 7.65(d, J = 8.0 Hz, 1H, Ar-H) 7.24 (d, J = 6.4 Hz, 1H, NH), 7.07 (d, J = 7.6 Hz, 2H, Ar-H), 7.01 (d, J = 8.4 Hz, 2H, Ar-H), 3.40 (q, J = 6.4 Hz, 1H, CH), 2.44 (d, J = 7.6 Hz, 2H, ArCH_2_), 1.85 (m, 1H, CH), 1.28 (d, J = 7.2 Hz, 3H, Me), 0.88 (dd, J = 6.6 Hz, 1.0 Hz, 6H, Me_2_); ^13^C NMR (100 MHz, CDCl_3_) δ 177.8, 143.4, 141.2, 136.4, 134.2, 132.8, 131.3, 130.8, 129.6, 128.5, 127.2, 44.9, 44.4, 30.2, 22.4, 22.3, 17.9; Anal. Calcd for C_19_H_22_ Cl_2_N_2_O_3_S: C 53.15, H 5.16, N 6.52. Found: C 53.16, H 5.18, N 6.54.

### *N*'-[(4-methoxyphenyl)sulfonyl]-2-[4-(2-methylpropyl)phenyl]propanehydrazide (4f)

Yield 94%, White solid; MP 96–98°C; IR (KBr) ν∼3315, 2953, 1673, 1597, 1154; ^1^H NMR (400 MHz, CDCl_3_) δ 7.82 (d, J = 6.4 Hz, 1H, NH), 7.68 (dt, J = 8.4 Hz, 3.2 Hz, 2.0 Hz, 2H, Ar-H), 7.24 (d, J = 6.4 Hz, 1H, NH), 7.07 (d, J = 7.6 Hz, 2H, Ar-H), 7.01 (d, J = 8.4 Hz, 2H, Ar-H), 6.84 (dt, J = 8.4 Hz, 3.2 Hz, 2.0 Hz, 2H, Ar-H), 3.84 (s, 3H, OMe), 3.40 (q, J = 6.4 Hz, 1H, CH), 2.44 (d, J = 7.6 Hz, 2H, ArCH_2_), 1.85 (m, 1H, CH), 1.28 (d, J = 7.2 Hz, 3H, Me), 0.88 (dd, J = 6.6 Hz, 1.0 Hz, 6H, Me_2_); ^13^C NMR (100 MHz, CDCl_3_) δ 177.8, 163.8, 141.2, 136.4, 130.8, 129.6, 127.2, 127.1, 114.1, 55.6, 44.9, 44.4, 30.2, 22.4, 22.3, 17.9; MS m/z 391 [M+H]; Anal. Calcd for C_20_H_26_ N_2_O_4_S: C 61.52, H 6.71, N 7.17. Found: C 61.54, H 6.73, N 7.18.

### Determination of reducing ability

Reducing ability of Ibuprofen and its derivatives (4a, 4b, 4c, 4d, 4e and 4f) along with quercetin as positive control were determined according to the method of Hsieh and Yan [22] with slight modifications. Briefly, samples (0–100 µM) were mixed with 2.5 mL of 200 mM sodium phosphate buffer, pH 6.6 containing 1% potassium ferricyanide and the mixture was incubated at 50°C for 20 min. At the end of incubation, 2.5 mL of 5% TCA was added and centrifuged at 450×*g* for 10 min. Further, 2.5 mL of supernatant was taken and mixed with 0.5 mL of aqueous 0.1% ferric chloride. The absorbance was measured at 700 nm against blank using UV/Vis spectrophotometer (BioMate 3S, Thermo scientifics).

### Antioxidant activity

The free radical scavenging activity of Ibuprofen and its derivatives (4a, 4b, 4c, 4d, 4e and 4f) along with quercetin as positive control were determined using 1, 1-diphenyl-2-picrylhdrazyl (DPPH) radical according to the method of Yamaguchi et al. [23] with slight modifications. Briefly, Samples (0–100 µM) were taken in test tubes with 1 mL of freshly prepared 0.1 mM DPPH solution and the final volume was made up to 2 mL using methanol. Samples were incubated for 20 min at room temperature in dark and the resulting absorbance was recorded at 517 nm against blank using UV/Vis spectrophotometer.

### Preparation of platelet-rich plasma and washed platelets

Venous blood was drawn from healthy drug-free human volunteers (non-smokers) approved by the Institutional Human Ethical Committee (IHEC-UOM No. 95/Ph.D/2013-14) University of Mysore, Mysore. Written consent were obtained from the healthy volunteers as per the guidelines of Institutional Human Ethical Committee (IHEC-UOM No. 95/Ph.D/2013-14). It was immediately mixed with acid citrate dextrose (ACD) anticoagulant (85 mM sodium citrate, 78 mM citric acid and 111 mM D-glucose) in the ratio 6∶1 (blood: ACD v/v). The anti-coagulated whole blood was then centrifuged at 90×*g* for 15 min and the supernatant thus obtained was the platelet-rich plasma (PRP). The PRP was centrifuged at 1,700×*g* for 15 min at 37°C. The platelet pellet thus obtained was suspended and incubated for 10 min in Tyrode's albumin buffer [145 mM NaCl, 5 mM KCl, 10 mM HEPES, 0.5 mM Na_2_HPO_4_, 1 mM MgCl_2_, 6 mM glucose, and 0.3% bovine serum albumin (BSA), pH 6.5] and washed thereafter at 1,700×*g* for 15 min at 37°C. The previous washing step was repeated one more time. Finally, the washed platelets were suspended in the Tyrode's albumin buffer (pH 7.4). The cell count was determined in both PRP and washed platelet suspension using a Neubauer chamber and adjusted to 5×10^8^ cells/ml in the final suspension using platelet poor plasma/Tyrode's albumin buffer (pH 7.4) [24].

### Determination of endogenously generated reactive oxygen species (ROS)

Endogenous ROS production in platelets was determined according to the method of Lopez et al. [25] with slight modifications using CMH2DCFDA, a ROS-sensitive fluorescent probe. PRP as well as washed platelet suspensions were independently treated with calcium ionophore (A23187, 10 µM) as agonist. For inhibition studies, pre-loaded platelets with agonist were incubated with 4f in increasing doses (0–100 µM) and the final volume was made up to 200 µL with HEPES-buffered saline [HBS, 145 mM NaCl, 10 mM HEPES, 10 mM D-glucose, 5 mM KCl, 1 mM MgSO_4_ and supplemented with 0.1% bovine serum albumin (BSA), pH 7.45] and incubated at 37°C for 1 h. The control (untreated) and treated platelets were then incubated with 10 µM CMH2DCFDA for 30 min at 37°C, fluorescence was recorded using Varioskan multimode plate reader (Thermo Scientifics, USA) by exciting the samples at 488 nm and measuring the resulting fluorescence at 530 nm.

### Estimation of intracellular calcium

Intracellular Ca^2+^ concentration was measured in PRP and washed platelets according to the method of Asai et al. [26] with slight modifications. Briefly, PRP and washed platelet suspensions were independently treated with A23187 (10 µM) as agonist. For inhibition studies, pre-loaded platelets with agonist were incubated with 4f in increasing doses (0–100 µM) and the final volume was made up to 200 µL with modified Tyrode's solution (150 mM NaCl, 2.7 mM KCl, 1.2 mM KH_2_PO_4_, 1.2 mM MgSO_4_, 1.0 mM CaCl_2_, 10 mM HEPES with 0.1% bovine serum albumin, pH 7.4) and incubated for 1 h at 37°C to induce the release of Ca^2+^ from the intracellular Ca^2+^ stores. Samples were then incubated for 45 min at room temperature with 2 µM fura-2/AM, a fluorescence Ca^2+^ indicator. The cells were subsequently washed twice with modified Tyrode's solution to remove the unbound dye and finally the platelet pellet was suspended in modified Tyrode's solution. The fura-2/AM absorption was determined by exciting the samples at 340 and 380 nm and the resulting fluorescence was measured at 500 nm. Data were presented as absorption ratios (340/380 nm).

### Assesment of cardiolipin peroxidation

NAO, a fluorescent probe was used to detect peroxidation of cardiolipin. NAO loses its affinity for peroxidised cardiolipin resulting in decreased fluorescence [27]. Both PRP and washed platelets were processed as discussed above with either A23187 (10 µM) as agonist or pre-loaded platelets with agonist were incubated with 4f in increasing concentrations (0–100 µM) for inhibition studies. After incubation, samples were loaded with NAO (5 µM) for 30 min at 37°C. After incubation fluorescence was recorded by exciting the samples at 499 nm and emission was recorded at 530 nm.

### Determination of changes in mitochondrial membrane potential (ΔΨ*m*)

Changes in ΔΨ*m* were determined using cationic dye, rhodamine 123 in which the intensity of the fluorescence decreases proportionally with decrease in ΔΨ*m* [28]. Both PRP and washed platelets were processed as discussed above with either A23187 (10 µM) as agonist or pre-loaded platelets with agonist were incubated with 4f in increasing concentrations (0–100 µM) for inhibition studies. After incubation, samples were then loaded with 0.2 µM rhodamine 123 followed by 15 min incubation at 37°C and the fluorescence was recorded by exciting the samples at 502 nm and the resulting emission was recorded at 527 nm.

### Assay of Caspases activity

PRP and washed platelets were processed as discussed previously with A23187 (10 µM) as agonist and 4f in increasing doses for inhibition studies along with A23187 as agonist. Platelet lysate was prepared by adding an equal volume of 2X Triton buffer (2% Triton X-100, 2 mM EGTA, 100 mM Tris-HCl - pH 7.2, 10 µg/mL leupeptin, 2 mM PMSF, 10 mM benzamidine, 2 mM Na_3_VO_4_) to the treated and control platelets and allowed to undergo lysis for 30 min at 4°C. The lysate was centrifuged at 16000×*g* for 5 min. The pellet thus obtained is the cytoskeleton-rich (Triton-insoluble) fraction, which was subjected to caspase activity.

Caspase activity was determined by incubating cell lysate in a microtitre plate with substrate solution (20 mM HEPES, pH 7.4, 2 mM EDTA, 0.1% CHAPS, 5 mM DTT and 8.25 µM caspase substrate (AC-DEVD-AMC for caspase-3 and AC-LEHD-AFC for caspase-9) for 2 h at 37°C. Substrate cleavage was measured with a multimode plate reader (excitation wavelength 360 nm and emission at 460 nm) [29].

### Determination of PS externalization

Both PRP and washed platelets were processed as discussed above with A23187 (10 µM) as agonist. For inhibition studies, pre-loaded platelets with agonist were incubated with 4f in increasing concentrations (0–100 µM) and incubated at 37°C for 1 h. Further, samples of treated and control PRP/washed platelets were transferred to equal volume of ice-cold 1% (v/v) glutaraldehyde in HBS for 10 min, and then incubated for 10 min with FITC labeled-annexin V (0.6 µg/mL) in HBS. The cells were collected by centrifugation for 60 s at 3000×*g* and resuspended in HBS. Cell staining was measured in a multimode plate reader by exciting the samples at 496 nm and emission was recorded at 516 nm [30].

### Detection of cytochrome c release

Cytochrome c release was detected by western blotting technique. Washed platelets (5×10^8^/ml) were treated with A23187 (10 µM) as agonist and for inhibition studies, pre-loaded platelets with agonist were incubated with 4f in increasing concentrations (0–100 µM) and incubated at 37°C for 1 h. Then the samples were lysed using freeze-thaw method whereby, cells were freezed for 10 min at −80°C and kept for 10 min at room temperature for thawing. The cycle was repeated for 4 times to get platelet lysate. Cytosolic and cytoskeleton fractions were separated by centrifugation at 10,500×*g* for 15 min. Cytosolic proteins were separated on SDS-PAGE (10%) and electroblotted on to PVDF membrane for 1 h at 50 V using wet blotter. Blots were then incubated overnight with 5% skimmed milk powder in tris-buffered saline with 0.1% tween 20 (TBST) to block residual protein binding sites. Later membrane was incubated with anti-cytochrome c antibody (1∶1,000) in TBST for 3 h followed by incubation with horseradish-peroxidase (HRP)-conjugated anti-IgG antibody (1∶10,000) in TBST. Blots were then developed by enhanced chemiluminescence method (ECL) [18] β-actin was used as loading control.

### Detection of protein tyrosine phosphorylation and caspase-3 by immunoblots

Washed platelets (5×10^8^/ml) were stimulated by adding collagen (1 µg/ml) as agonist for protein tyrosine phosphorylation, A23187 (10 µM) as agonist for caspase-3 activation along with different doses of 4f (0–100 µM) for inhibition studies. After 3 min of incubation for protein tyrosine phosphorylation and 1 h for caspase-3 activation, platelet suspensions were lysed by adding 10 µL of HEPES buffer containing 10% SDS, 20 mM N-ethylmaleimide, 20 mM sodium *o*-vanadate, 50 mM EDTA and 10 mM PMSF. Following centrifugation, the supernatants were separated on SDS-PAGE (10%) and electroblotted on to a PVDF membrane. After blocking with 5% skimmed milk powder in TBST, the blots were probed with monoclonal anti-phosphotyrosine antibody (1∶1,000) for protein tyrosine phosphorylation and anti-caspase-3 antibody for caspase-3 in TBST for 3 h. Blots were then incubated with horseradish-peroxidase (HRP)-conjugated anti-IgG antibody (1∶10,000) in TBST and blots were finally developed by ECL method [24] β-actin was used as loading control.

### Determination of cytotoxicity by MTT assay

MTT [3-(4,5-dimethylthiazol-2-yl)-2,5-diphenyltetrazolium bromide] colorimetric assay was performed to assess cell viability. PRP was taken separately in polystyrene 96-well microtiter plates and treated with A23187 (10 µM) as agonist and 4f (0–100 µM) for inhibition studies along with agonist and the final volume was made up to 200 µL with HBS. After 1 h of incubation 250 µM of MTT was added and incubated for additional 3 h. Thereafter, MTT was removed and remaining formazan crystals were completely dissolved in DMSO. Afterwards, the absorbance was recorded at 570 nm, using Varioskan multimode plate reader [31].

### Measurement of LDH leakage

PRP was treated with different doses of 4f (0–100 µM) along with A23187 (10 µM) as agonist for 1 hr. Following, platelets were pelleted by centrifugation at 1700×*g* for 10 min. Supernatants were used to detect LDH release by kit method, according to the manufacturer's protocol. The assay was performed in a time course of decrease in NADH absorbance at 340 nm for 3 min.

### Measurement of γ-glutamyltransferase (GGT) activity

To determine the GGT activity, washed platelets were suspended in tubes with 100 µL HBS, each containing 2×10^6^ platelets/µL. Further, platelets were treated with A23187 (10 µM) as agonist and for inhibition studies 4f in increasing concentrations (0–100 µM) along with agonist and incubated for 1 h. After incubation, platelets were pelleted, suspended in distilled water and lysed by sonication. The resulting lysate was used to determine GGT activity. Platelet-GGT activity was done according to the method of Sener et al. [32], which included assay mixture containing 4 mM γ-glutamyl *p*-nitroanilide and 40 mM glycylglycine in 185 mM Tris-HCl buffer, pH 8.2. The results were calculated using molar extinction coefficient of *p*-nitroanilide (9,900 M^−1^cm^−1^) at 405 nm and expressed as mM *p*-nitroanilide formed/min/mg protein.

### Platelet aggregation

Platelet aggregation was determined by turbidimetric method with a dual channel Chrono-log model 700-2 aggregometer (Havertown, USA). Briefly, 250 µL of PRP were taken in siliconized glass cuvette and pre-incubated for 3 min at 37°C with different concentrations of compound 4f (0–100 µM), and the aggregation was initiated by the addition of collagen (2 µg/mL)/ADP (10 µM)/epinephrine (10 µM). The aggregation was then followed with constant stirring at 1200 rpm for 6 min [24].

### Platelet adhesion assay

Platelet adhesion assay was carried out according to the method of Bellavite et al. [33]. Briefly, collagen was immobilized on to 96-well polystyrene microtiter plates by adding 20 µg of collagen type I in 200 µL phosphate buffered saline (PBS, 10 mM, pH 7.4) to each well and left overnight at 4°C. Following which, 200 µL of 1% (w/v) BSA in PBS was added to block the wells and incubated at 37°C for 1 h. The wells were then washed three times with PBS. In the first set of experiment, 4f (0–100 µM) was directly added to the collagen-coated wells, pre-incubated for 10 min, washed three times with PBS and then PRP was added. In the second set of experiment, PRP pre-treated with 4f (0–100 µM) for 10 min at 37°C was added to the collagen-coated wells. Total reaction volume was made up to 200 µL with PBS. The reaction mixture was incubated at 37°C for 90 min and then washed three times with PBS. The adherent platelets were then lysed with 150 µL lysis buffer (100 mM citrate buffer pH 5.4 containing 5 mM *p*-nitrophenyl phosphate and 0.1% Triton X-100) at 37°C for 90 min. The reaction was terminated by inactivating the platelet membrane acid phosphatase activity with the addition of 100 µL stopping reagent (2 N NaOH). The color developed was measured at 405 nm. Platelet adhesion was expressed as percent adhesion, considering PBS-treated platelet suspension as 100%.

### Protein estimation

Protein estimation was carried out according to the method of Lowry et al. [34] using BSA as standard.

### Statistical analysis

Results were expressed as mean ± SEM of five independent experiments. Statistical significance among groups was determined by one way analysis of variance (ANOVA) followed by Tukey's test for comparison of mean [n = 5, *p*<0.05 (^#^), *p*<0.01 (**/^##^), *p*<0.001 (***/^###^); *: significant compared to control platelets and #: significant compared to A23187 treated platelets].

## Results

### Synthesis of new Ibuprofen derivatives

**Figure 1:**
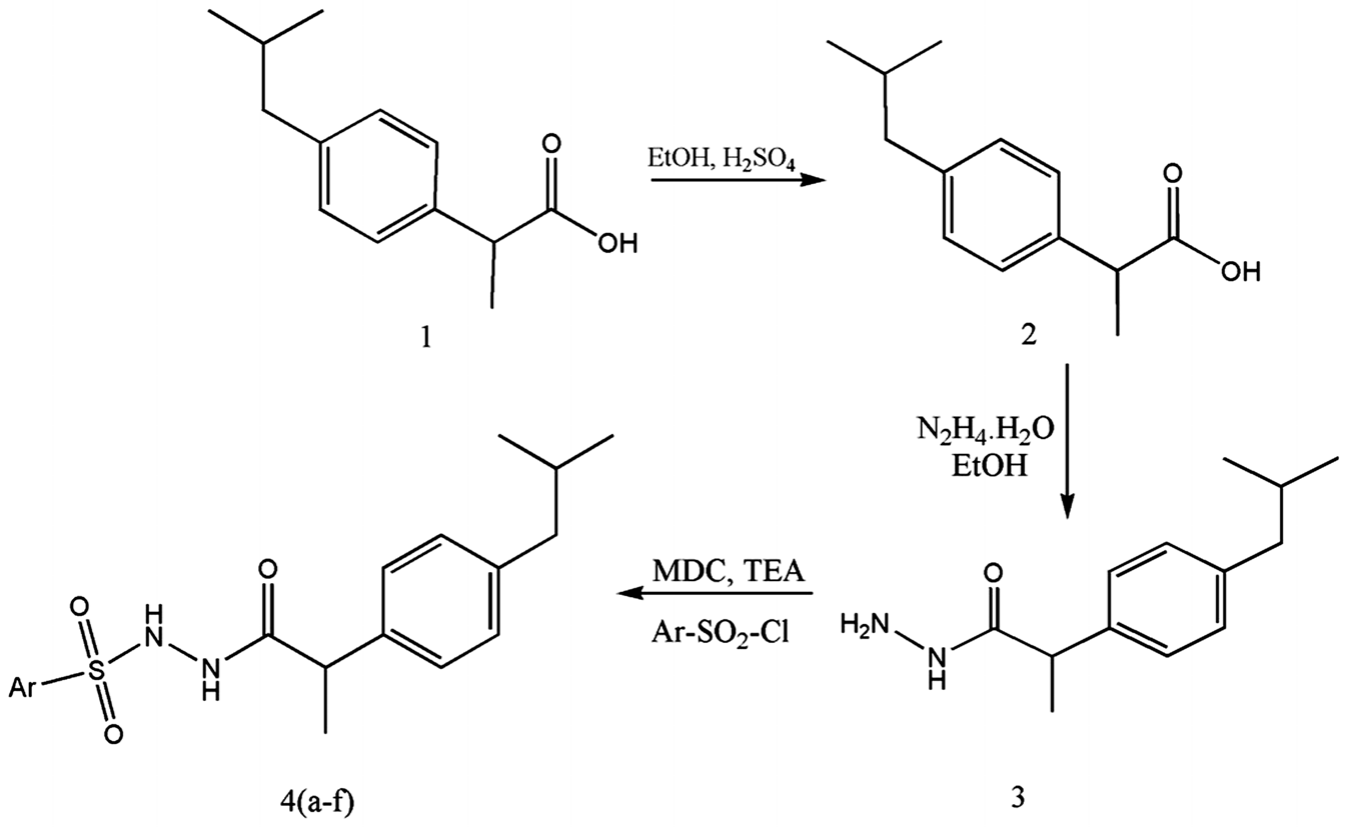
Reaction sequence for the synthesis of new Ibuprofen derivatives.

The novel derivatives of Ibuprofen 1 described in this study, were prepared by the reaction sequence as shown in **Fig. 1**. Esterification of parent compound (Ibuprofen) using ethanol in presence of sulfuric acid furnished ethyl 2-[4-(2-methylpropyl)phenyl]propanoate. Upon refluxing the resulting ester 2 with alcoholic hydrazine hydrate gave 2-(4-isobutylphenyl)propanehydrazide (3). Further, sulfonation of 3 with various sulfonyl chlorides generated N'-(2-(4-isobutylphenyl)propanoyl)-4-methoxybenzenesulfonohydrazide 4(a-f). The synthesized compounds were chemically characterized (**Table 1**).

**Table 1:** Physical and chemical characteristics of new Ibuprofen derivatives.

Entry	Ibuprofen derivatives	Yield (%)	Melting point (°C)	Molecular formula	Molecular weight
4a	2-[4-(2-methylpropyl)phenyl]-N'-(phenylsulfonyl)propanehydrazide	89	112–114	C_19_H_24_N_2_O_3_S	360
4b	N'-[(4-fluorophenyl)sulfonyl]-2-[4-(2-methylpropyl) phenyl]propanehydrazide	91	121–123	C_19_H_23_FN_2_O_3_S	378
4c	N'-[(4-bromophenyl)sulfonyl]-2-[4-(2-methylpropyl)phenyl]propanehydrazide	81	116–118	C_19_H_23_BrN_2_O_3_S	438
4d	N'-[(4-nitrophenyl)sulfonyl]-2-[4-(2-methylpropyl)phenyl]propanehydrazide	78	142–144	C_19_H_23_N_3_O_5_S	405
4e	N'-[(2,5-dichlorophenyl)sulfonyl]-2-[4-(2-methylpropyl)phenyl]propanehydrazide	85	102–104	C_19_H_22_Cl_2_N_2_O_3_S	428
4f	N'-[(4-methoxyphenyl)sulfonyl]-2-[4-(2-methylpropyl)phenyl]propanehydrazide	94	96–98	C_20_H_26_N_2_O_4_S	390

### Free radical scavenging potential of Ibuprofen and its derivatives

**Figure 2:**
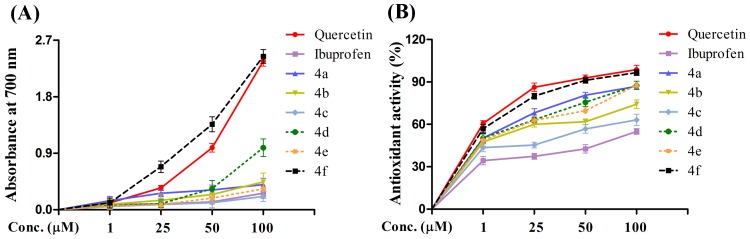
Efficacy of Ibuprofen and its derivatives measured in terms of (A) Reduction potential (B) DPPH radical scavenging activity in comparison with Quercetin. Values are presented as mean ± SEM (n = 5).

Determination of anti-oxidant properties of Ibuprofen and its derivatives prior to the evaluation of their anti-apoptotic properties is essential, as molecules with strong anti-oxidant properties are ideally preferred in combating against oxidative stress induced platelet apoptosis. Thus, Ibuprofen and its derivatives (4a-4f) were evaluated for their anti-oxidant properties. To begin with, reducing ability of Ibuprofen and its derivatives on Fe^3+^ to Fe^2+^ was evaluated by ferric reducing power assay. It was observed that the compound 4f had significant and dose dependently increasing reducing ability (**Fig. 2A**). Further, DPPH radical scavenging potential of Ibuprofen and its derivatives were also evaluated. Compound 4f showed marked radical scavenging potential owing it to be a potent anti-oxidant molecule compared with other derivatives of Ibuprofen (**Fig. 2B**). Based on these results, compound 4f was proved to possess strong anti-oxidant property and appeared to be promising for the evaluation of its anti-apoptotic nature and platelet aggregation inhibitory properties. Quercetin is being used as a standard anti-oxidant compound to compare the efficacy of test molecules.

### Compound 4f alleviates A23187 induced ROS and intracellular calcium levels

**Figure 3:**
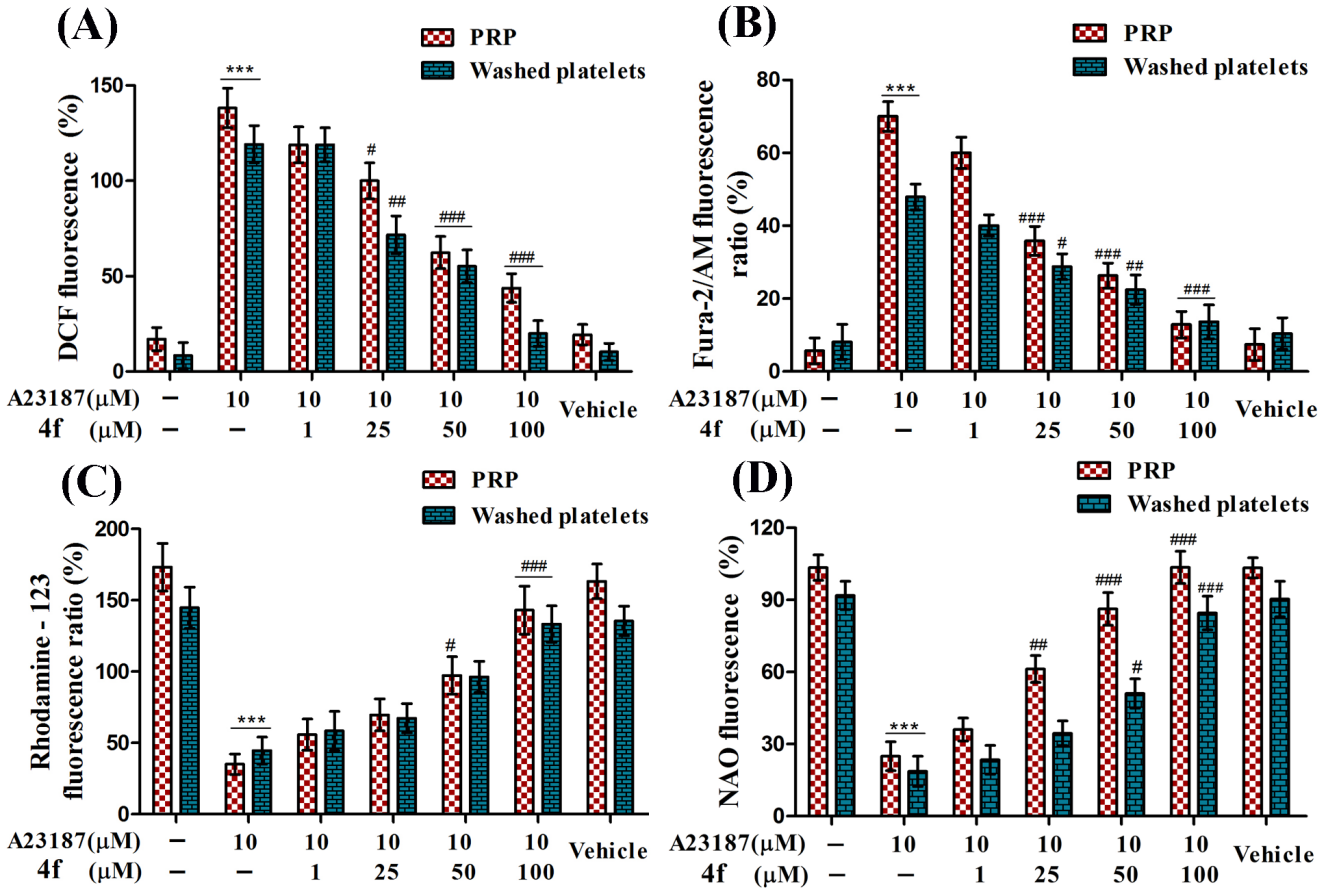
Effect of compound 4f on A23187 induced (A) Endogenous generation of ROS (B) Intracellular calcium levels (C) Mitochondrial membrane depolarization and (D) Peroxidation of cardiolipin in PRP and washed platelets. Values are presented as mean ± SEM (n = 5), expressed as percentage increase in DCF fluorescence (for ROS), percentage increase in Fura-2/AM fluorescence (intracellular calcium), Rhodamine 123 fluorescence (ΔΨ*m*) and NAO fluorescence (cardiolipin), relative to control. ***p<0.001; significant compared to control. ^#^ *p*<0.05, ^##^ *p*<0.01, ^###^ *p*<0.001; significant compared to A23187.

Platelets being anuclear generally undergo apoptosis *via* intrinsic pathway mediated by mitochondria where mitochondrial generated ROS play imperative role in triggering apoptosis. Therefore, in order to investigate the anti-apoptotic properties of compound 4f, it is ideal to analyze the ROS scavenging properties of 4f. Platelets treated with standard agonist A23187, evoked significant ROS levels with 138% in PRP and 119% in washed platelets respectively (**Fig. 3A**). Compound 4f was able to significantly reduce A23187 evoked ROS in a concentration dependent manner with marked reduction of ROS levels was observed at 50 and 100 µM concentrations. Platelets undergoing oxidative stress are associated with elevated levels of intracellular calcium which drives platelets towards intrinsic apoptotic pathway. Consequently, 4f was scrutinized for its effects on dwindling intracellular calcium levels in platelets where, A23187 was used as an agonist in increasing intracellular calcium concentration in platelets. Compound 4f was able to reduce A23187 induced increase in intracellular calcium in a dose dependent manner and significant reduction was observed at 100 µM in both PRP and washed platelets (**Fig. 3B**).

### Effect of compound 4f on ΔΨ*m* depolarization and peroxidation of cardiolipin

As mitochondria are the primary sources of ROS production, making them highly susceptible to ROS attacks which includes dissipation of ΔΨ*m* and peroxidation of cardiolipin which is a major component of mitochondrial membrane. Therefore, 4f was evaluated for its efficacy in preventing ΔΨ*m* depolarization and cardiolipin peroxidation. A23187 was used as agonist in inducing ΔΨ*m* depolarization which dissipated ΔΨ*m* significantly. Besides, 4f markedly restored dissipation of ΔΨ*m* in a concentration dependent manner and at 100 µM concentration there was complete restoration of ΔΨ*m* to basal levels (**Fig. 3C**). Further, 4f was assessed for its effects on cardiolipin peroxidation with A23187 as agonist which showed significant increase in cardiolipin peroxidation. 4f which was proved to be potent in reducing oxidative stress in the previous set of experiments also showed marked abrogation of A23187 induced peroxidation of cardiolipin in a concentration dependent manner. At 100 µM concentration of 4f there was complete inhibition of cardiolipin peroxidation (**Fig. 3D**).

### Arresting of cytosolic cytochrome c expression by compound 4f

**Figure 4:**
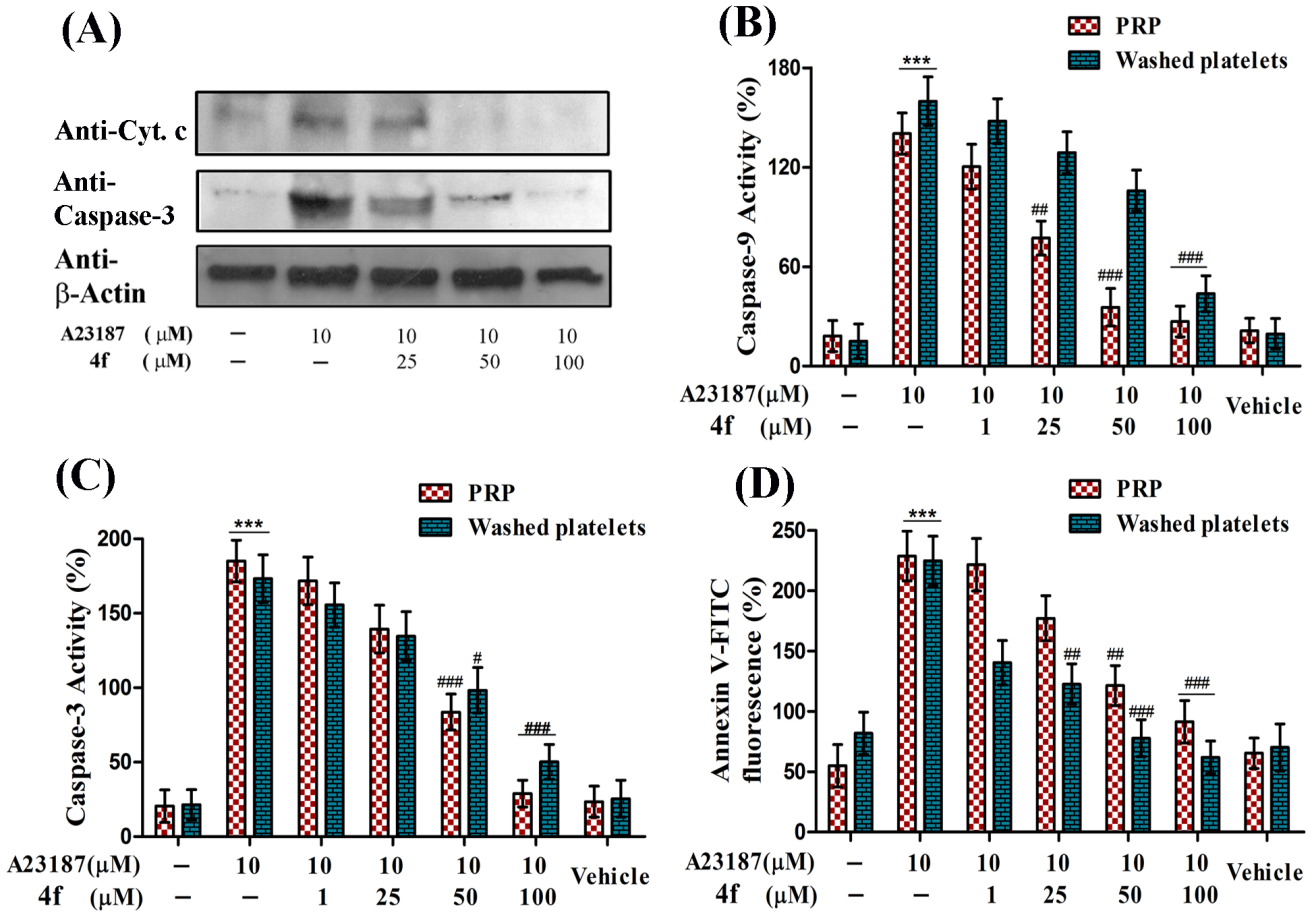
Effect of compound 4f on A23187 induced (A) Translocation of cytosolic cytochrome C and activation of caspase-3 (B) Caspase-9 and (C) Caspase-3 activities and (D) PS externalization in PRP and washed platelets. Values are presented as mean ± SEM (n = 5), expressed as percentage increase in (B & C) caspase activity and (D) Annexin V-FITC fluorescence and expressed as percentage increase in apoptotic platelets expressing PS relative to control. ***p<0.001; significant compared to control. ^#^ *p*<0.05, ^##^ *p*<0.01, ^###^ *p*<0.001; significant compared to A23187.

Release of cytochrome c from mitochondria to cytosol is mediated by the formation of MPTP. Altered redox conditions and peroxidation of cardiolipin play critical role in the formation of MPTP and the release of cyt c into the cytosol. A23187 was used as agonist in inducing the release of cyt c to the cytosol. On the other hand, 4f was also able to diminish the expression of cytosolic cyt c induced by A23187 in a dose dependent manner and at 100 µM concentration basal levels of cyt c was restored (**Fig. 4A**).

### Inhibition of caspase activity by compound 4f

Intrinsic apoptotic pathway in principally is mediated by caspase-9 which is activated by itself bound to apoptosome complex along with cytosolic cyt c. The activated caspase-9 in turn activates caspase-3 which orchestrates platelet apoptosis. Therefore, in order to investigate the anti-apoptotic properties of 4f, inhibition of caspase-9 and caspase-3 activity is critically important. Compound 4f which was proved to be effective in ameliorating oxidative stress, was able to significantly inhibit both caspase-9 and caspase-3 activities induced by A23187 in a concentration dependent manner (**Fig. 4B** & **Fig. 4C**). Further, inhibition of caspase-3 was confirmed by western blot wherein, there was significant reduction in activated form of caspase-3 in platelets treated with 4f (**Fig. 4A**).

### Mitigation of PS externalization by compound 4f

Cells undergoing apoptosis are finally marked by the scrambling of PS from inner plasma membrane to outer plasma membrane. A23187 was used as agonist in inducing PS externalization in platelets and its inhibition by 4f was evaluated. As expected, there was dose dependent inhibition of PS externalization by 4f in a concentration dependent fashion and significant inhibition was observed at 100 µM concentration in both PRP and washed platelets (**Fig. 4D**).

### Suppression of protein phosphorylation by compound 4f

**Figure 5:**
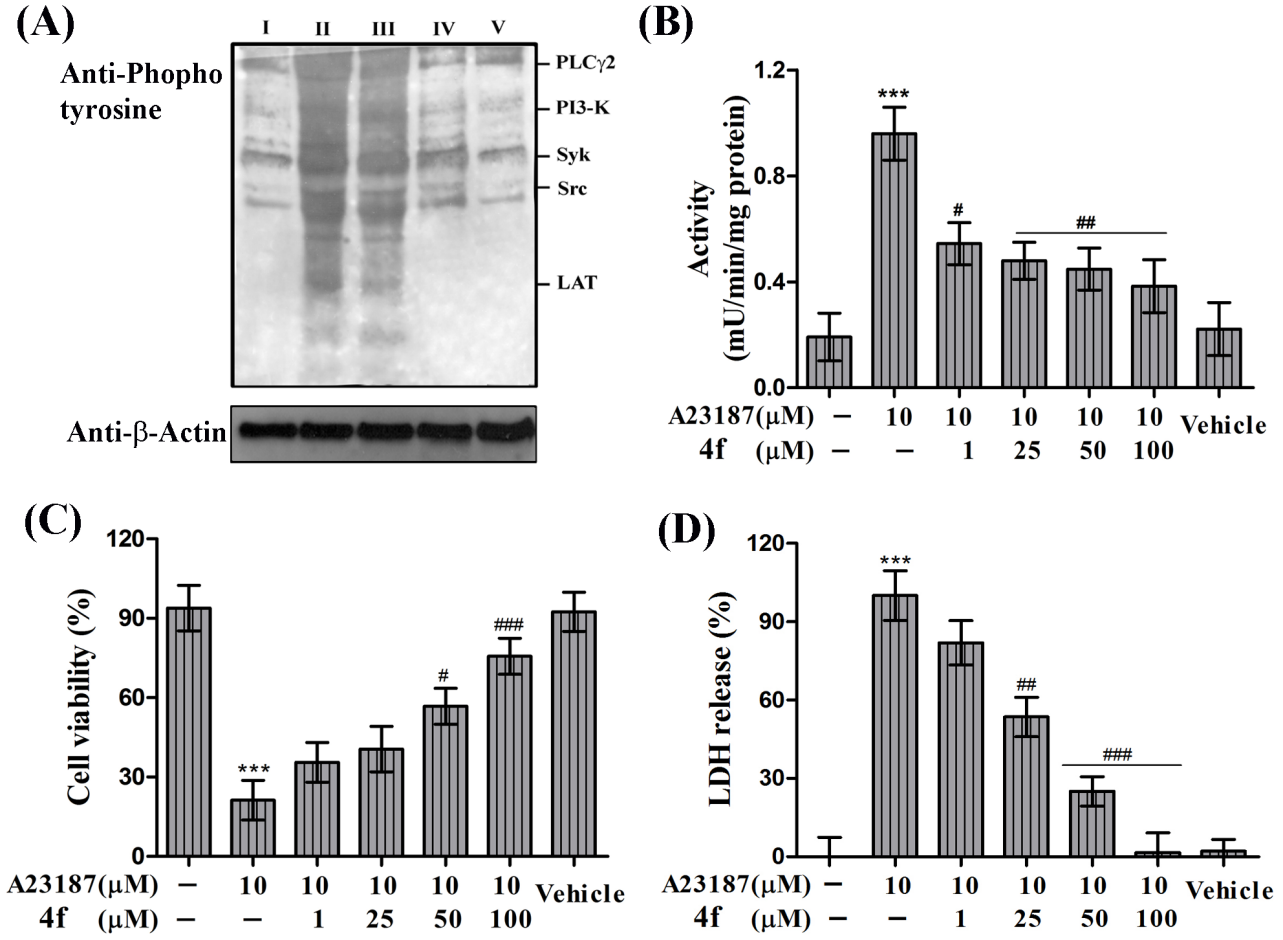
Effect of compound 4f on (A) Collagen induced protein phosphorylation (B) A23187 induced γ-glutamyltransferase activity (C) MTT cell viability assay (D) LDH release in platelets. (A) Lane I- resting platelets (untreated). Lane II- platelets treated with Collagen (1 µg/mL). Lanes III, IV and V- pre-loaded platelets with collagen and incubated with 4f in increasing concentration of 25, 50 and 100 µM respectively. Values are presented as mean ± SEM (n = 5). ***p<0.001; significant compared to control. ^#^ *p*<0.05, ^##^ *p*<0.01, ^###^ *p*<0.001; significant compared to agonist.

Recently, it has been revealed that sites for protein phosphorylation are exposed by caspase cleavage activity. Therefore, 4f was evaluated for its effects on protein phosphorylation with collagen as agonist. There was significant increase in protein phosphorylation in platelets treated with collagen whereas, 4f markedly reduced collagen induced protein phosphorylation in a concentration dependent manner and at 100 µM concentration protein phosphorylation was restored up to basal levels (**Fig. 5A**).

### Inhibition of γ-glutamyl transferase (GGT) activity by compound 4f

GGT is involved in GSH homeostasis and its expression is increased during oxidative stress. In the previous set of experiments 4f was proved to be anti-apoptotic by reducing oxidative stress in the platelets. Accordingly, 4f was analyzed for its effects on GGT activity. There was significant increase in GGT activity in platelets treated with A23187 as agonist and as expected there was concentration dependent reduction of GGT activity in agonist activated platelet suggesting that 4f serves as anti-apoptotic molecule basically by reducing oxidative stress in platelets (**Fig. 5B**).

### Cyto-protective effects of compound 4f

In order to investigate whether 4f had cyto-protective effect, MTT assay was performed. A23187 was used as standard agonist, which reduced cell viability from 93% to 21%. However, treating platelets with 4f restored its survivability up to 75% in agonist activated platelets (**Fig. 5C**). Further, cyto-protective nature of 4f was confirmed by measure the leakage of LDH into the medium. Treatment of platelets with A23187 increased LDH release in to the medium whereas, 4f was able to significantly reduce LDH release indicating its cyto-protective properties (**Fig. 5D**).

### Effect of compound 4f on agonist induced platelet aggregation and platelet adhesion

**Figure 6:**
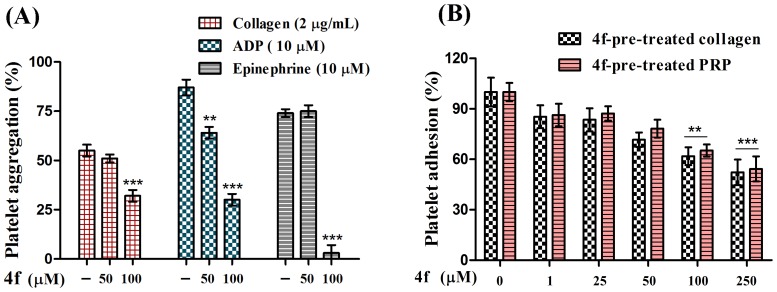
Effect of compound 4f on (A) Platelet aggregation induced by Collagen/ADP/Epinephrine and (B) Platelet adhesion on immobilized collagen type I with compound 4f pre-treated collagen and PRP pre-treated with compound 4f. Values are presented as mean ± SEM (n = 5), expressed as percentage decrease in aggregation and increase in platelet adhesion. **p<0.01, ***p<0.001; significant compared to control.

Further, platelets being the key mediators in maintaining the integrity of endothelium, effect of compound 4f on platelet aggregation and platelet adhesion with collagen was evaluated. In order to stimulate platelet aggregation collagen/ADP/epinephrine were used as agonist. Compound 4f was able to significantly inhibit agonist induced platelet aggregation to varied extent. Of all the agonists tested, the epinephrine-induced aggregation was abolished at 100 µM concentration of compound 4f. On the other hand, compound 4f alone did not exhibit any effect on platelet aggregation up to the tested concentrations (**Fig. 6A** **& Fig. S1–S3**).

In order to determine the effect of compound 4f on the platelet receptors for agonists, collagen adhesion was performed using pre-coated collagen microtiter wells. The compound 4f untreated/PBS treated platelet suspension served as control and accounted for 100% adhesion. In contrast, 4f pre-treated platelets did not adhere efficiently to collagen immobilized on the microtiter wells. Compound 4f was able to prevent the binding of platelet with collagen in a concentration dependant fashion in both collagen pre-treated with compound 4f and PRP pre-treated with compound 4f. At 250 µM concentration, only 54% adhesion was observed, and thus affecting the collagen binding property of platelets (**Fig. 6B**).

## Discussion

Reduced platelet count or thrombocytopenia is the medical condition where the platelet count drops drastically to 50,000/µL of blood as against the normal count of 150,000 to 450,000 platelets/µL of blood. It can be caused by a variety of clinical conditions including 1) decreased production of platelets or 2) augmented platelet destruction or 3) increased splenic sequestration of platelets. Among these reasons, augmented platelet destruction or apoptosis can be seen in a number of medical and pathological conditions [1], [4], [35]. The platelet apoptosis is mediated either by immune related or non-immune related causes. The immune thrombocytopenia is a common bleeding disorder characterized by autoantibody-mediated platelet destruction. The auto-antibodies primarily target the membrane receptors and integrin complex of platelets including glycoproteins GPIIb/IIIa and GPIb/IX [36]. In contrast, platelets undergo oxidative stress-mediated apoptosis in non-immune related thrombocytopenia. The adverse effects of thrombocytopenia may lead to morbidity and mortality from severe surgical hemorrhage and delay in the normal process of clotting. Several studies reported oxidative stress or immunologic reaction-induced platelet apoptosis by a wide range of biologicals including antibiotics, anticancer drugs, phytochemicals and hormones [5], [8], [9]. Therefore, the clinical implication of thrombocytopenia in various human pathologies has strongly demanded the need for small molecular weight natural/synthetic platelet protective molecules. Till date only two phytochemicals (cinnamtannin B1 and crocin) are shown to inhibit platelet apoptosis [17], [18]. Hence, there continues the search for potent small molecular weight natural/synthetic molecules to manage thrombocytopenia associated with various human pathologies.

Of late, Ibuprofen, a well known NSAID was reported to interfere with platelet functions and viability thereby, causing thrombocytopenia, nevertheless it is also demonstrated to have anti-cancer, analgesic, anti-pyretic and anti-platelet properties [19], [37], [38]. Thus, as an attempt to reduce ibuprofen side effects including platelet damage property, in the present study we have synthesized a series of ibuprofen derivatives (4a-f) with improved antioxidant activity. Among the derivatives, compound 4f was a potent antioxidant and effectively inhibited oxidative stress-induced apoptosis in platelets.

In the first set of experiments, the effect of compound 4f on oxidative stress was assessed. A23187 was used to stimulate the endogenous generation of ROS in platelets. When the platelets were pretreated with 4f prior to A23187 treatment, the ROS generation was found to decrease in a dose-dependent manner. It is reported that it is specifically H_2_O_2_ that initiates the apoptotic events in platelets through the intrinsic or mitochondrial pathway. Thus, the results highlight the ability of compound 4f to diminish oxidative stress in platelets and thereby protect them from undergoing an early death. This property may be attributed to the anti-inflammatory and anti-platelet efficacy of the parent compound ibuprofen. Previously it has been reported that ibuprofen treatment decreases the levels of lipid peroxidation, tyrosine nitration, protein oxidation and ROS production in murine model of Alzheimer's disease [39].

For the next set of experiments, A23187 was used as the agonist to induce the different events of apoptosis. In order to assess the effect of compound 4f on mitochondria, the central players in the intrinsic pathway of apoptosis, changes in ΔΨ*m* were analyzed. A23187 was used as the positive control to induce changes in ΔΨ*m*. Compound 4f markedly diminished A23187-induced changes in ΔΨ*m* and reinstates the membrane potential. From the result it can be stated that 4f has the capacity to inhibit platelet apoptosis by protecting mitochondria from oxidative stress. In the recent past, a study by Sanz-Blasco et al. [40] demonstrated that ibuprofen prevents mitochondrial Ca^2+^ overload, cyt c release and thus prevent neurotoxicity [40]. Thus, to verify whether the same was true in the case of the effect of 4f on platelets, the levels of intracellular Ca^2+^ and cytosolic cyt c were measured. It was found that compound 4f could inhibit the increase in intracellular Ca^2+^ and cytosolic cyt c levels in a dose-dependent manner. Release of cyt c from mitochondrial intermembrane space to the cytosol due to formation of a channel MPTP is a key event that initiates morphological changes during apoptosis. The morphological changes are arbitrated by the activated caspases, finally resulting in PS externalization [7], [8], [15].

Therefore, the next set of experiments targeted the influence of compound 4f over caspase activity and PS exposure. The results of the study firmly highlight the inhibitory effect of compound 4f on the activities of caspase -9 and -3. Further, it was also shown that compound 4f was to impair PS exposure, the hallmark of an apoptotic cell. Thus, the current study underscores anti-apoptotic effects of compound 4f on human platelets. It was recently reported that the parent compound possesses chemo-preventive property *via* the stimulation of apoptosis [21], [41]. Besides, it was also reported that it causes thrombocytopenia, which is a common effect of most of the anti-cancer drugs [21]. Consequently, it would be amazing if compound 4f could wield anti-cancer effect. It has the potential to be developed as an anti-cancer molecule, which has platelet-protective properties.

Furthermore, effect of compound 4f on platelet aggregation was evaluated to delineate its cardio-protective action. Platelet aggregation plays a central role in the perpetuation of CVDs and atherothrombotic disorders. It triggers intraluminal thrombosis and thereby promulgates myocardial infarction, stroke and peripheral vascular occlusions [42], [43]. Thus, inhibition of platelet aggregation is crucial in prevention of CVDs and associated complications. The results demonstrated that, compound 4f significantly inhibited various agonists (Collagen/ADP/Epinephrine)-induced platelet aggregation to varied extent. Among the agonists, compound 4f showed better inhibition towards the epinephrine-induced platelet aggregation. The role of compound 4f in blocking epinephrine receptor on platelets is not clear. The receptors for the above-mentioned agonists are high molecular weight glycoproteins and compound 4f might interfere with the binding of agonists to receptors by interacting directly with the membrane proteins or membrane lipids. In continuance, platelet adhesion assay was carried out to verify whether compound 4f prevents the interaction of platelets with collagen. This interaction is crucial for platelet aggregation. The results indicate that compound 4f markedly decreases the collagen-binding efficacy of platelets, suggesting the mechanism through which the compound exerts its antiplatelet effect.

It has been reported that ibuprofen has mild anti-platelet effect, which is very less when compared to the other standard anti-platelet drugs such as aspirin and ketoprofen. However, from the current results the derivative of ibuprofen, compound 4f has been demonstrated to be a very effective anti-platelet agent. Aspirin is the most common anti-platelet agent used in the primary prevention of cardiovascular events due to atherothrombosis. Aspirin inhibits the platelet aggregation by reducing the production of thromboxane (Tx) A_2_ due to inhibition of cyclooxygenase-1 in platelets [44]. This could be the possible reason for aspirin-associated bleeding during chronic intake of aspirin. In addition, aspirin could also aggravate platelet GP Ib and GPV ectodomain shedding, which is yet another possible mechanism for hemorrhagic conditions [45]. Recent reports suggested the apoptotic tendency of aspirin toward many cell types including platelets [46]. Zhao et al. [47] demonstrated pro-apoptotic nature of aspirin on platelets and found that aspirin (2.5–20 mM) induced apoptosis in platelets via COX-independent pathway [47]. This could be the possible mechanism for aspirin induced hemorrhage in susceptible patients. In contrast, the compound 4f inhibited the agonist-induced platelet aggregation as well as protected the platelets from oxidative stress-induced apoptosis.

Platelets are known to express the amyloid precursor protein (APP) and exhibit the complete enzymatic machinery to process APP proteins into amyloid-β (Aβ) peptides through the same pathway described in the brain. A recent study demonstrated the amyloid-β (Aβ) peptide-induced platelet apoptosis by assessing ROS generation, increase of cytosolic Ca^2+^ levels, mitochondrial depolarization, caspase-3 activation, cell shrinkage and cell membrane scrambling. In neurological pathologies, uncontrolled activation of platelets by oxidative stress and other inducers can lead to acute vessel occlusion leading to myocardial infarction and stroke [3]. In this context, platelet protective and anti-aggregant molecules like compound 4f, crocin and cinnamtannin B1 [17], [18] could be used in the treatment regime of neurological disorders as auxiliary therapeutic molecules in order to mitigate the platelet apoptosis and altered hemostasis.

Taken together, the therapeutic drug-induced dysregulated platelet apoptosis is one of the major causes for thrombocytopenia. Furthermore, the formation of MPs during platelet apoptosis might worsen the clinical condition [5]. Platelet-derived MPs (PMPs) are shown to regulate several pathophysiological functions including cell proliferation, differentiation, vascular remodeling, angiogenesis, inflammation and apoptosis. The augmented levels of circulating PMPs have shown to be associated with many pathological conditions including CVDs, thromboembolism, diabetes, rheumatoid arthritis, and cancer [5], [16]. Therefore, biologicals with anti-platelet property as well as platelet protective efficacy stand better and thus could receive a lot of attention by the medical practitioners in order to treat thrombolytic disorders. From the results of the present study, it can be noted that compound 4f (100 µM) is as effective as cinnamtannin B1 and crocin, the previously reported compounds with platelet protective properties at the same concentration [17], [18]. Thus, compound 4f can be taken into account as a potential candidate in the treatment regime of altered platelet function-associated pathologies. In addition, compound 4f can be used as an auxiliary therapeutic agent in order to mitigate the side effects of therapeutic drugs especially platelet apoptosis and microparticle generation. Future studies related to the molecular mechanism of platelet protection and aggregation inhibition of compound 4f is highly exciting.

## Supporting Information

Figure S1 **Effect of compound 4f on Collagen induced platelet aggregation.** Concentration dependent inhibition of collagen induced platelet aggregation by compound 4f: (i) Control (Collagen-2 µg/mL), (ii) 50 µM and (iii) 100 µM compound 4f respectively and aggregation was performed as described in materials and methods section.
(TIF)

Figure S2 **Effect of compound 4f on ADP induced platelet aggregation.** Concentration dependent inhibition of ADP induced platelet aggregation by compound 4f: (i) Control (ADP-10 µM), (ii) 50 µM and (iii) 100 µM compound 4f respectively and aggregation was performed as described in materials and methods section.
(TIF)

Figure S3 **Effect of compound 4f on epinephrine induced platelet aggregation.** Concentration dependent inhibition of epinephrine induced platelet aggregation by compound 4f: (i) Control (Epinephrine-10 µM), (ii) 50 µM and (iii) 100 µM compound 4f respectively and aggregation was performed as described in materials and methods section.
(TIF)
