# Correction: A New Ibuprofen Derivative Inhibits Platelet Aggregation and ROS Mediated Platelet Apoptosis

**DOI:** 10.1371/journal.pone.0114675

**Published:** 2014-11-24

**Authors:** The PLOS ONE Staff

Figure 1 is incorrect. The authors have provided a corrected version here.

**Figure 1:**
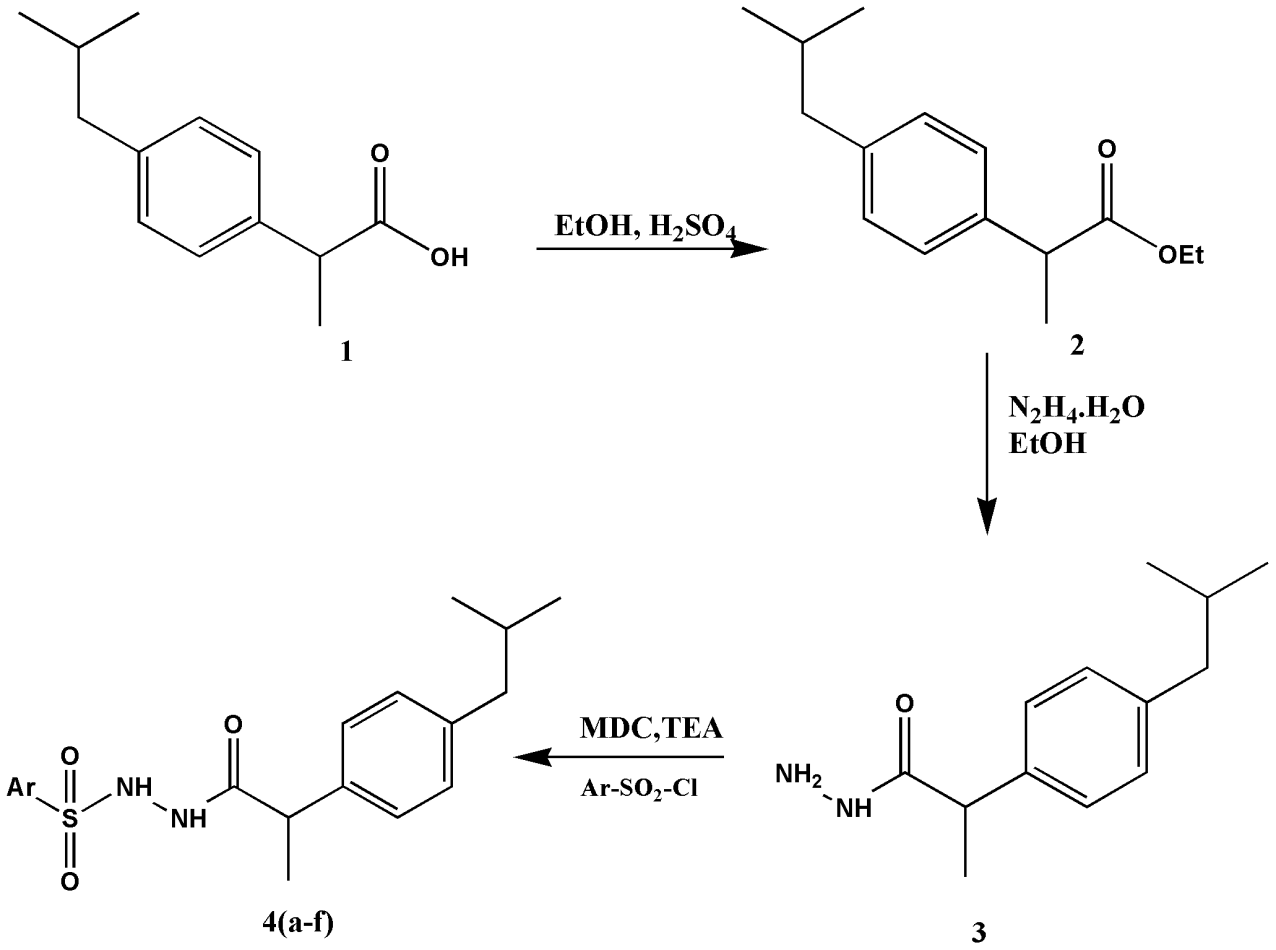
Reaction sequence for the synthesis of new Ibuprofen derivatives.
