# Supplementary figures and images for: A New Ibuprofen Derivative Inhibits Platelet Aggregation and ROS Mediated Platelet Apoptosis
Source: PLoS One. 2014 Sep 19;9(9):e107182. doi: 10.1371/journal.pone.0107182 (PMC4169656; doi:10.1371/journal.pone.0107182)

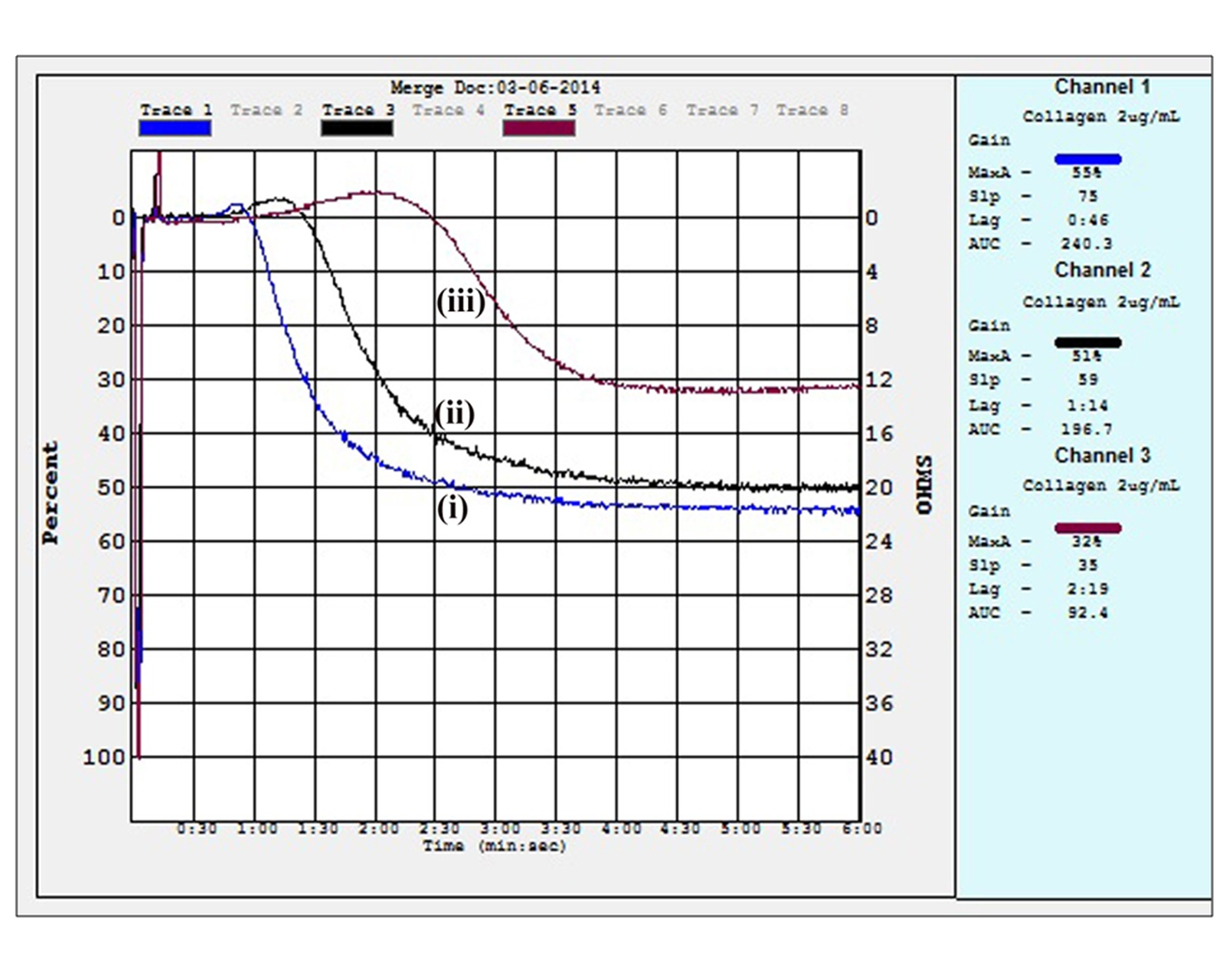

Supplement: Figure S1 — Effect of compound 4f on Collagen induced platelet aggregation. Concentration dependent inhibition of collagen induced platelet aggregation by compound 4f: (i) Control (Collagen-2 µg/mL), (ii) 50 µM and (iii) 100 µM compound 4f respectively and aggregation was performed as described in materials and methods section. (TIF) [file pone.0107182.s001.tif]

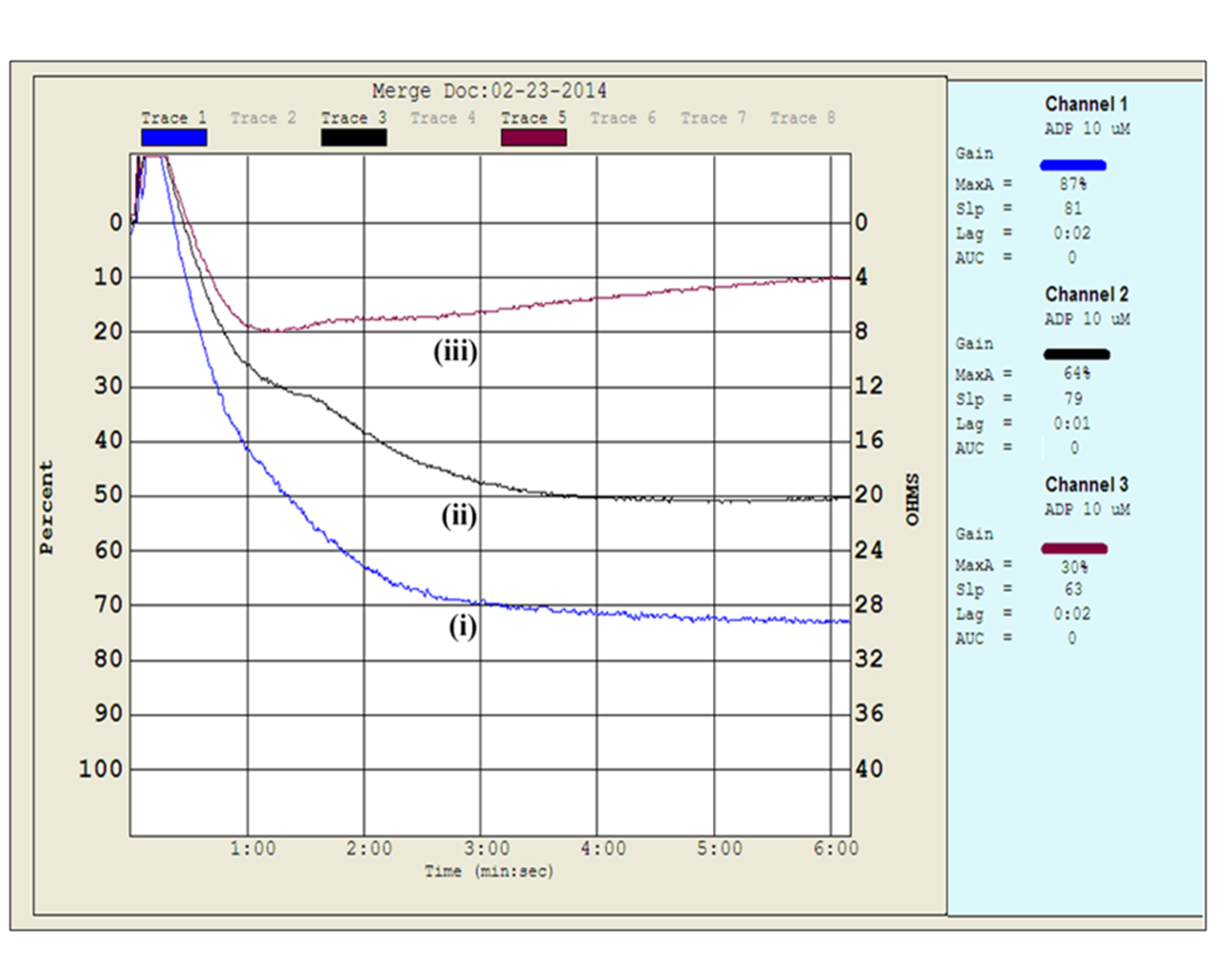

Supplement: Figure S2 — Effect of compound 4f on ADP induced platelet aggregation. Concentration dependent inhibition of ADP induced platelet aggregation by compound 4f: (i) Control (ADP-10 µM), (ii) 50 µM and (iii) 100 µM compound 4f respectively and aggregation was performed as described in materials and methods section. (TIF) [file pone.0107182.s002.tif]

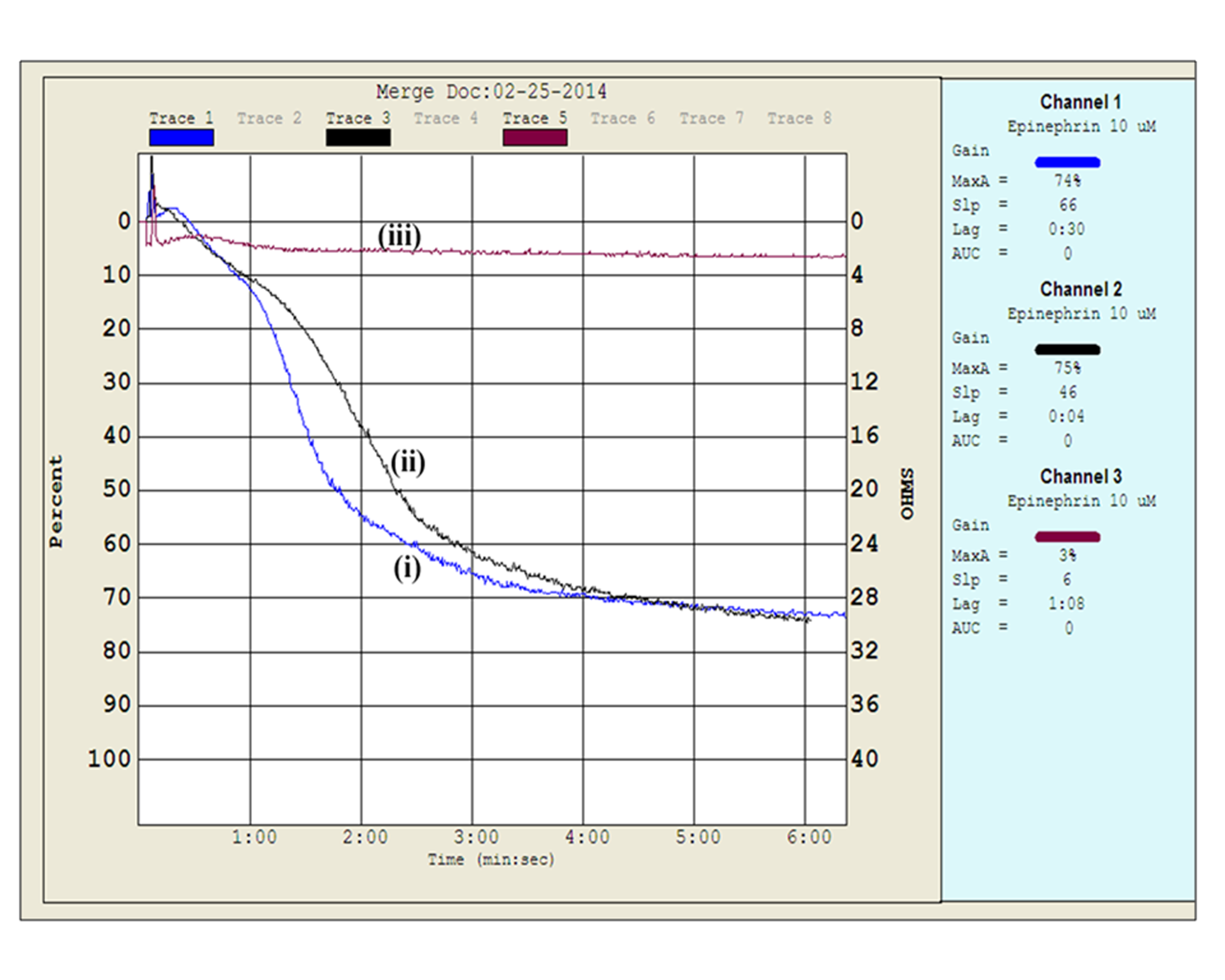

Supplement: Figure S3 — Effect of compound 4f on epinephrine induced platelet aggregation. Concentration dependent inhibition of epinephrine induced platelet aggregation by compound 4f: (i) Control (Epinephrine-10 µM), (ii) 50 µM and (iii) 100 µM compound 4f respectively and aggregation was performed as described in materials and methods section. (TIF) [file pone.0107182.s003.tif]
